# Transcriptional meta-analysis and immune cell profiles reveal altered neutrophil dynamics in chronic atrial fibrillation

**DOI:** 10.1098/rsos.241102

**Published:** 2025-03-05

**Authors:** Elijah Stone, Jude Taylor, Amy Li, Craig S. McLachlan

**Affiliations:** ^1^ Centre for Healthy Futures, Torrens University Australia, Surry Hills, New South Wales 2010, Australia; ^2^ Department of Rural Clinical Sciences, La Trobe University – Bendigo Campus, Bendigo, Victoria 3552, Australia; ^3^ School of Medical Sciences, The University of Sydney – Camperdown and Darlington Campus, Sydney, New South Wales 2006, Australia

**Keywords:** atrial fibrillation, progression, immune cells, neutrophil, angiogenesis, gene expression meta-analysis

## Abstract

Atrial fibrillation (AF) can present as persistent or permanent forms with each exhibiting distinct pathological features. This study explores the gene signatures associated with cardiac and immune cells in persistent and permanent AF compared to sinus rhythm controls. We performed a meta-analysis to combine independent microarray and RNA sequencing (RNAseq) datasets for both persistent and permanent AF left atrial tissue. Cell type abundances were inferred using Cibersort and CibersortX, and gene set enrichment analysis was performed using ShinyGo. In persistent AF, a significant reduction in atrial cardiomyocytes and smooth muscle cells, along with an increase in fibroblasts, myeloid cells and pericytes was observed. Permanent AF showed increased endothelial cell and pericyte abundance. Immune cell analysis revealed altered abundances in five cell types in persistent AF, particularly an increase in neutrophils, which was not observed in permanent AF. Pathway analysis identified enriched neutrophil activation and degranulation in persistent AF, while permanent AF was enriched in extracellular matrix organization and angiogenesis pathways. In conclusion, this study highlights distinct and complex immune and cellular dynamics between chronic AF, where persistent AF has heightened immune cell infiltration and neutrophil activity which contribute to sustaining AF, whereas permanent AF shows inflammation returning to baseline but enhanced tissue remodelling and angiogenesis.

## 1. Introduction

Atrial fibrillation (AF) is a heart rhythm abnormality that can be characterized by irregular and often rapid heart rate, which can lead to serious complications such as stroke, heart failure and thromboembolism. AF is classified as paroxysmal, persistent and permanent primarily based on the duration of each AF episode [1]. Paroxysmal AF occurs intermittently and resolves within 7 days without treatment. Reoccurring AF beyond a week suggests progression to persistent AF and will typically require treatment to regulate heart rate [2]. Permanent AF maintains AF rhythm beyond 12 months that does not resolve with treatment but is rather managed symptomatically [3]. AF can result from both structural and electrical remodelling [4]; however, there is translational evidence to suggest that structural remodelling is an important predictor of persistent/permanent AF.

Neutrophils are the most abundant innate immune cells that act as first responders during inflammation. In AF, structural remodelling of the atrial tissue, influenced by ongoing inflammation and immune responses, plays a crucial role in the persistence of the arrhythmia and progression of the disease [5]. Several studies have highlighted the need for different treatment approaches to permanent AF [2,6,7]. Standard approaches such as pulmonary vein isolation in permanent AF were shown to be suboptimal and further suggest that the pathology for permanent AF may be different from that of persistent AF. The remodelling process is also associated with angiogenesis, the formation of new blood vessels, which is promoted by inflammatory cells like neutrophils. These cells contribute to the pathological changes, but the need remains to better understand the pathological drivers between persistent and permanent AF.

This complexity is further underscored by the differential expression of NOX2, a member of the NADPH oxidase family, which plays a significant role in oxidative stress and inflammation [8]. NOX2 expression varies between acute and permanent AF, with elevated levels of soluble NOX2 in serum being associated with acute onset AF and acute pneumonia [9]. In patients with paroxysmal or persistent AF, NOX2 upregulation leads to increased production of isoprostanes, which are eicosanoids involved in cardiac inflammation and immunity. However, this upregulation is not observed in permanent AF, indicating a potential difference in the inflammatory pathways involved [10,11].

Despite these insights, the precise immune cell populations and regulatory pathways driving the differences between persistent and permanent AF remain unclear. This study aims to investigate the temporal expression of immune cells, particularly neutrophils, in atrial tissue using a meta-analysis of multiple open-source RNA sequencing (RNAseq) and microarray datasets. The advantage of this approach lies in its ability to increase statistical power and robustness by integrating data from multiple independent studies, thereby providing a more comprehensive understanding of gene expression patterns. By revealing differences in immune cell infiltration and gene activity, our study sheds light on the pathological characteristics that may differentiate persistent versus permanent AF. The findings from our study will provide a better understanding of the disease mechanisms that may be used to inform more effective treatment strategies.

## 2. Material and methods

### 2.1. Microarray and RNAseq datasets

The Affymetrix Human Genome U133 Plus 2.0 microarray datasets GSE41177, GSE115574, GSE79768, GSE14975 and GSE2240 and the Illumina NextSeq 5000 RNAseq dataset GSE128188 were identified in the Gene Expression Omnibus (GEO) and selected based on the inclusion of left atrial myocardial tissue samples from AF patients and sinus rhythm (SR) controls. All samples were obtained from valve repair surgery or coronary artery bypass grafting. Samples of SR were from patients with no evidence of clinical AF in the absence of antiarrhythmic drugs. The raw microarray CEL files or RNAseq count matrices were downloaded from the GEO and imported into R (https://www.r-project.org/) for subsequent analysis using R and Bioconductor (https://www.bioconductor.org/). RNAseq sequence files were aggregated by SR control and aligned to the GRC human genome build 38 using STAR and gene count matrices were generated using Stringtie and the R package IsoformSwitchAnalyseR for subsequent analysis.

### 2.2. Microarray and RNAseq data analysis

The software platform R (v. 3.4) and the Bioconductor packages compatible with the Affymetrix microarray platforms for quality control, probe annotation and filtering, and statistical analysis were utilized; affy, limma (lmFit). The CEL files were quality assessed and then processed using affy, Brainarray custom chip description and probe re-annotation files for the U133 Plus 2.0 array (http://brainarray.mbni.med.umich.edu). The differential log_2_-transformed probe intensities between AF tissue samples and control tissue samples were determined using lmFit (limma), and a Benjamini–Hochberg adjusted *p*-value of less than 0.05 was considered significant. The R package edgeR was used for bulk RNAseq quality control, filtering and statistical analysis to identify differentially expressed genes (DEGs) between groups.

To meta-analyse gene expression profiles of AF subtypes across independent datasets, random-effects meta-analysis was performed using the R package MetaVolcanoR. Based on the random-effects model, an average fold-change expression and summary *p*-value of each gene were obtained, and those with a combined random *p* < 0.05 and concordance designated as sign consistency |SigCon| ≥ 3 between datasets were considered significant. SigCon indicates whether a gene consistently shows the same direction of change across multiple studies, e.g. SigCon of −3 means that the gene is consistently downregulated in all three datasets. Persistent AF datasets included GSE41177, GSE115574 and GSE79768. Permanent AF datasets included GSE14975, GSE2240 and GSE128188. Venn diagrams of DEGs were generated using https://bioinformatics.psb.ugent.be/webtools/Venn/. Meta-volcano plots were generated using MetaVolcanoR, gplots, ggplot and RColorBrewer packages in R.

For further identification of pathway-specific genes, gene set enrichment of concordant significantly DEGs from meta-analysis was done using the online Web platform ShinyGo v. 0.80 (http://bioinformatics.sdstate.edu/go/). Kyoto encyclopedia of genes and genomes (KEGG) and Gene Ontology biological pathway (GO BP) databases were used to identify enriched gene pathways with an FDR cutoff of 0.05. The top 20 pathways in each database were sorted by fold enrichment and then by −log_10_(FDR), and plotted as stem plots using ShinyGo.

### 2.3. Cibersort and CibersortX cellular deconvolution

Cibersort employs a core matrix of 547 genes and machine learning to robustly deconvolute 22 lymphoid and myeloid lineages from gene expression data, including naive B cells, memory B cells, plasma cells (or plasmablasts), cytotoxic T cells (CD8), helper T cells (CD4 naive, memory and follicular), regulatory CD4 T cells, gamma-delta T cells, natural killer cells (NK cells; resting and activated), dendritic cells (resting and activated), monocytes, macrophages (M0, M1 and M2), mast cells (resting and activated), eosinophils and neutrophils [12]. Cibersort was run using a gene expression matrix derived from the Affymetrix microarray datasets following instructions on the Cibersort website (https://cibersort.stanford.edu/). Cibersort calculates each of the 22 leukocyte populations as a fraction of a total of one for each individual sample.

Similarly, CibersortX was used to deconvolve cell signatures specific to atrial tissue. The cellular signatures were generated from combined left and right atrial cell counts from the reference atlas (human heart cell atlas; https://www.heartcellatlas.org/) using the Seurat R package [13,14]. The cell-specific pseudo-bulk gene expression profiles were then used to generate a signature matrix and applied using CibersortX to deconvolute cardiac cell type proportions based on gene expression. Note that one permanent AF dataset was excluded from the CibersortX analysis as the sample size was too low. The relative expression of myocardial cell types, namely adipocytes, atrial cardiomyocytes, endothelial cells, fibroblasts, lymphoid cells, myoendothelial cells, myeloid cells, neuronal cells, pericytes and smooth muscle cells, was quantified.

### 2.4. Statistical analysis

Data are expressed as means and standard deviations unless indicated otherwise. Statistical and graphed analyses comparing Cibersort and CibersortX data between AF and SR groups were generated using GraphPad Prism v. 9.3.1. Unpaired *t*‐test was used to compare immune cells and cardiac cells between groups and *p* < 0.05 was considered statistically significant. All other graphical visuals were generated using R.

## 3. Results

### 3.1. Differential gene expression from meta-analysis

**Figure 1.**
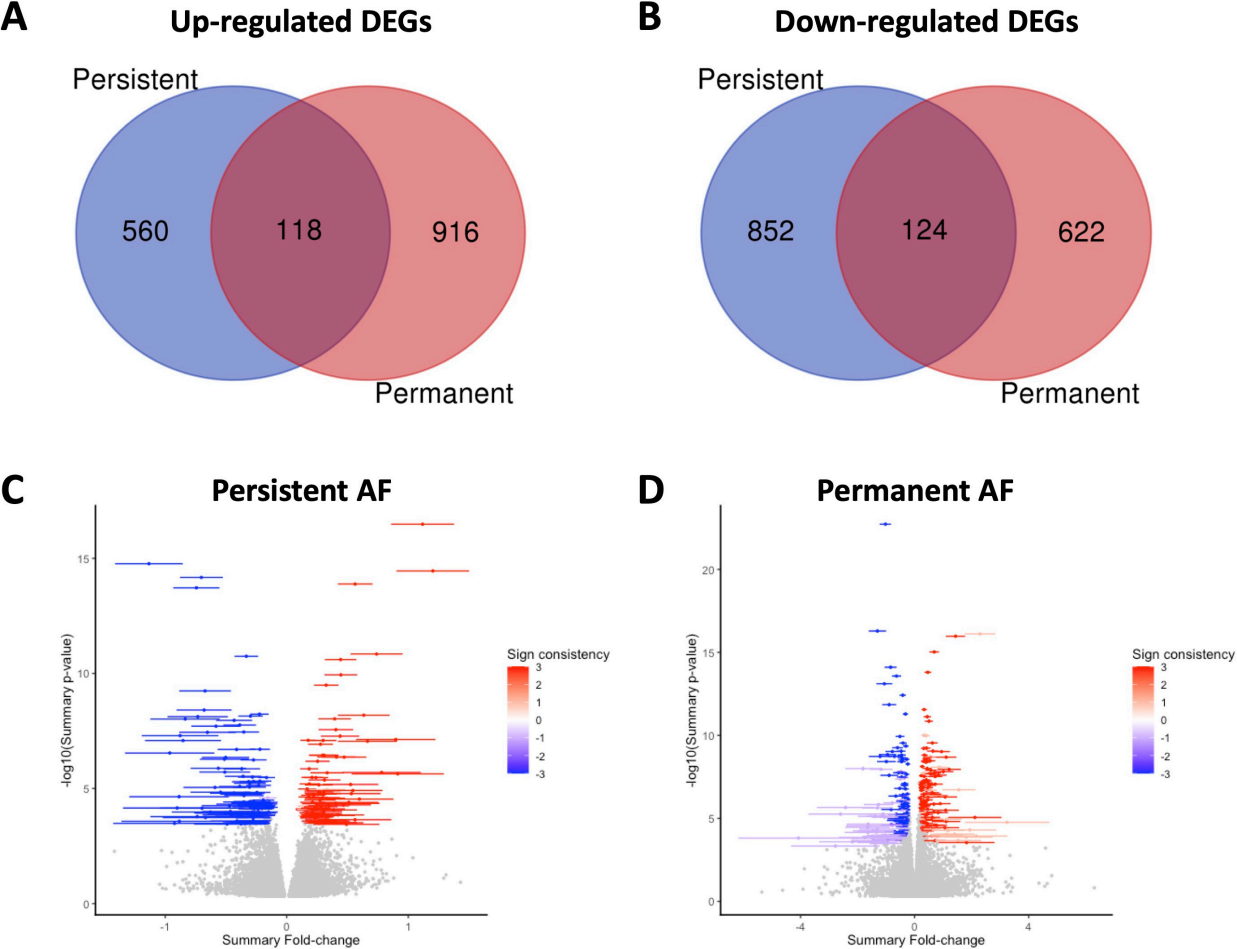
Differentially expressed genes (DEGs). (*A,B*) Venn diagram showing the number of DEGs in the persistent (blue) and permanent (red) AF groups that are upregulated (*A*) and downregulated (*B*). *(C,D*) Meta-volcano plots illustrating the confidence interval of each DEG. Sign consistency indicates whether a gene consistently shows the same direction of change across multiple studies.

A meta-analysis was performed to compare the gene expression of three independent datasets of persistent AF and three independent datasets of permanent AF to their respective SR controls. DEGs were identified by *p* < 0.05, and their consistent expression across all grouped datasets. In persistent AF, 1674 DEGs were identified, where 678 genes and 976 genes were respectively upregulated and downregulated (figure 1A,B; electronic supplementary material, table S1). In permanent AF, 1780 DEGs were identified with 1034 genes and 746 genes respectively upregulated and downregulated (figure 1A,B; electronic supplementary material, table S2). The variability of each DEG in both persistent AF (figure 1C) and permanent AF (figure 1D) groups is illustrated by the meta-volcano plots. These DEGs were applied to all subsequent pathway analyses.

### 3.2. Inferred cell type analysis

**Figure 2.**
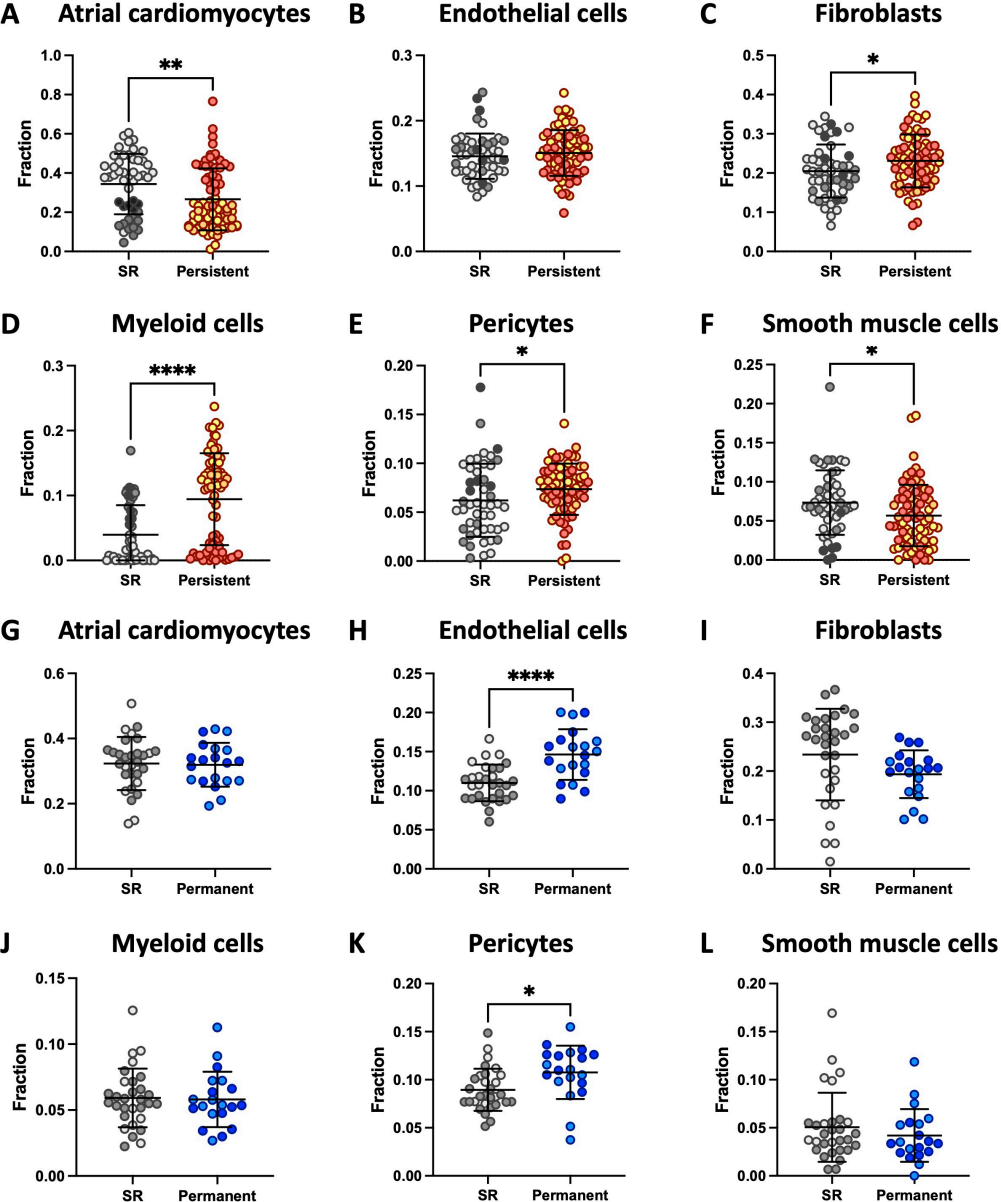
Imputation of cell-type-specific gene expression from (*A–F*) persistent AF and (*G–L*) permanent AF patients by CibersortX. Persistent AF (*n* = 74), permanent AF (*n* = 20) and their respective sinus rhythm (SR) controls (*n* = 49 for persistent, *n* = 30 for permanent) from atrial samples are compared. Samples from each dataset are grouped by colour. Significance is denoted as *p < 0.05, **p < 0.01 and ****p < 0.0001.

CibersortX was used to determine cell type abundances expressed as a fraction of a total of one for each individual sample. Atrial-specific cell type gene signatures were generated from a single-cell human heart reference dataset [14]. In persistent AF, the overall abundance of atrial cardiomyocytes (figure 2A) and smooth muscle cells (figure 2F) was reduced while an increase in fibroblasts (figure 2C), myeloid cells (figure 2D) and pericytes (figure 2E) was identified compared to SR controls. An increase in endothelial cell (figure 2H) and pericyte (figure 2K) abundance was present in permanent AF that was significant relative to SR controls. The abundances of other cell types, including adipocytes, lymphoid cells, myoendothelial cells and neuronal cells, were not different between AF and control groups.

### 3.3. Immune cell type analysis

**Figure 3.**
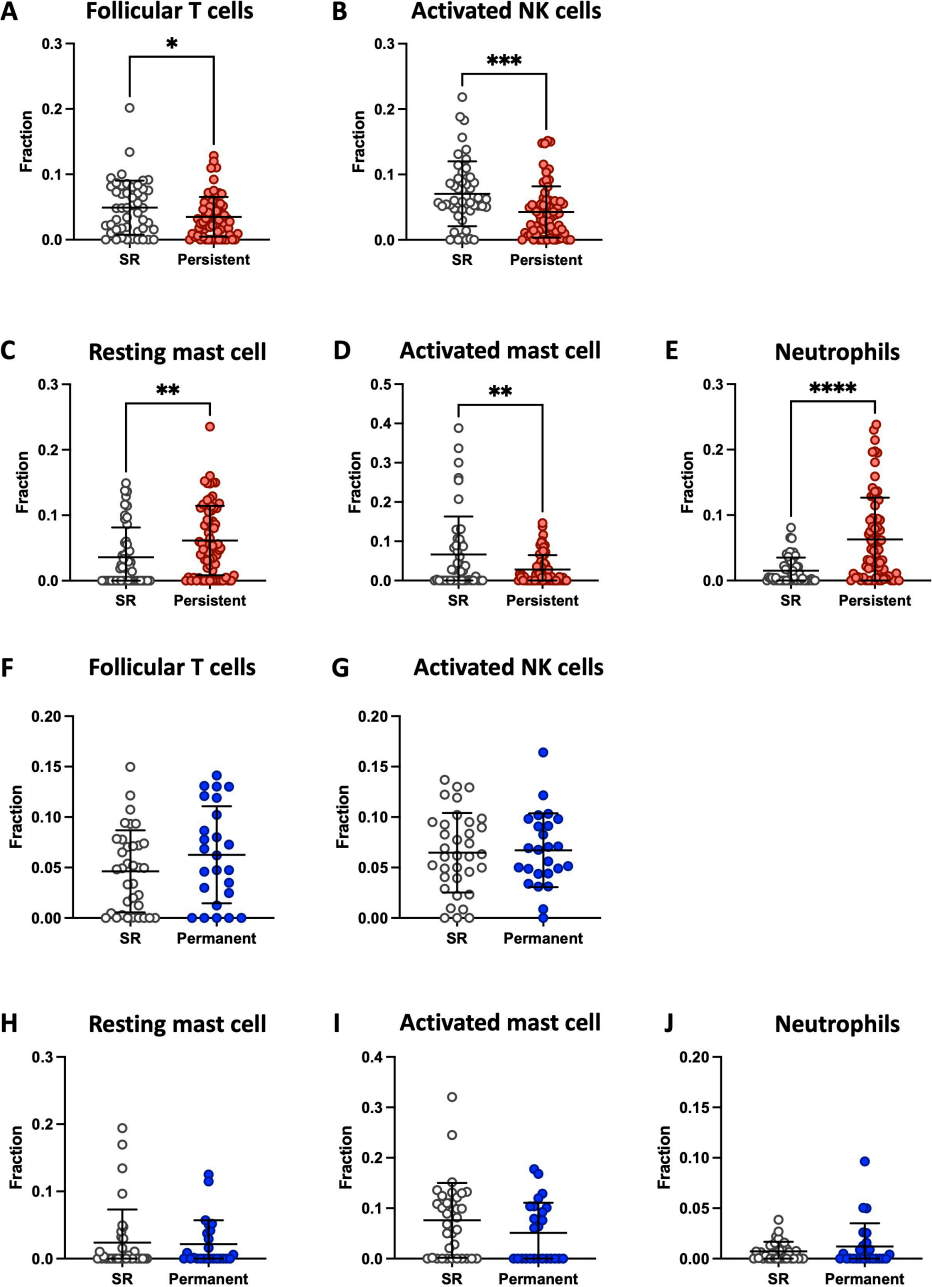
Immune cell deconvolution. (A–E) Persistent AF (*n* = 74), (F–J) permanent AF (*n* = 25) and their respective sinus rhythm (SR) controls (*n* = 49 for persistent, *n* = 35 for permanent) from atrial samples are compared. (A,F) Follicular T cells, (B,G) activated NK cells, (C,H) resting mast cells, (D,I) activated mast cells, and (E,J) neutrophils. Cibersort immune cell fractions were determined for each sample, where each data point represents an individual sample. Significance is denoted as *p < 0.05, **p < 0.01, ***p < 0.001 and ****p < 0.0001.

Since myeloid cell abundance in persistent AF differed from SR controls, we examined the expression of immune cell infiltrates using Cibersort. Immune cell abundances were significantly altered in persistent AF compared to SR controls across five cell types (figure 3A–E) including follicular T cells, NK cells, mast cells and neutrophils. In contrast, these same cell types were not significantly different in permanent AF from SR controls (figure 3F–J). Plasma cell levels were elevated in persistent AF (*p* = 0.039) and naive B cells were elevated in permanent AF (*p* = 0.036) compared to SR controls (data not shown). The abundances of other cell types were not different between AF and control groups, which include memory B cells, cytotoxic T cells (CD8), helper T cells (CD4 naive, memory and follicular), regulatory CD4 T cells, gamma-delta T cells, resting NK cells, dendritic cells (resting and activated), monocytes, macrophages (M0, M1 and M2) and eosinophils.

### 3.4. Gene set enrichment analysis

**Figure 4.**
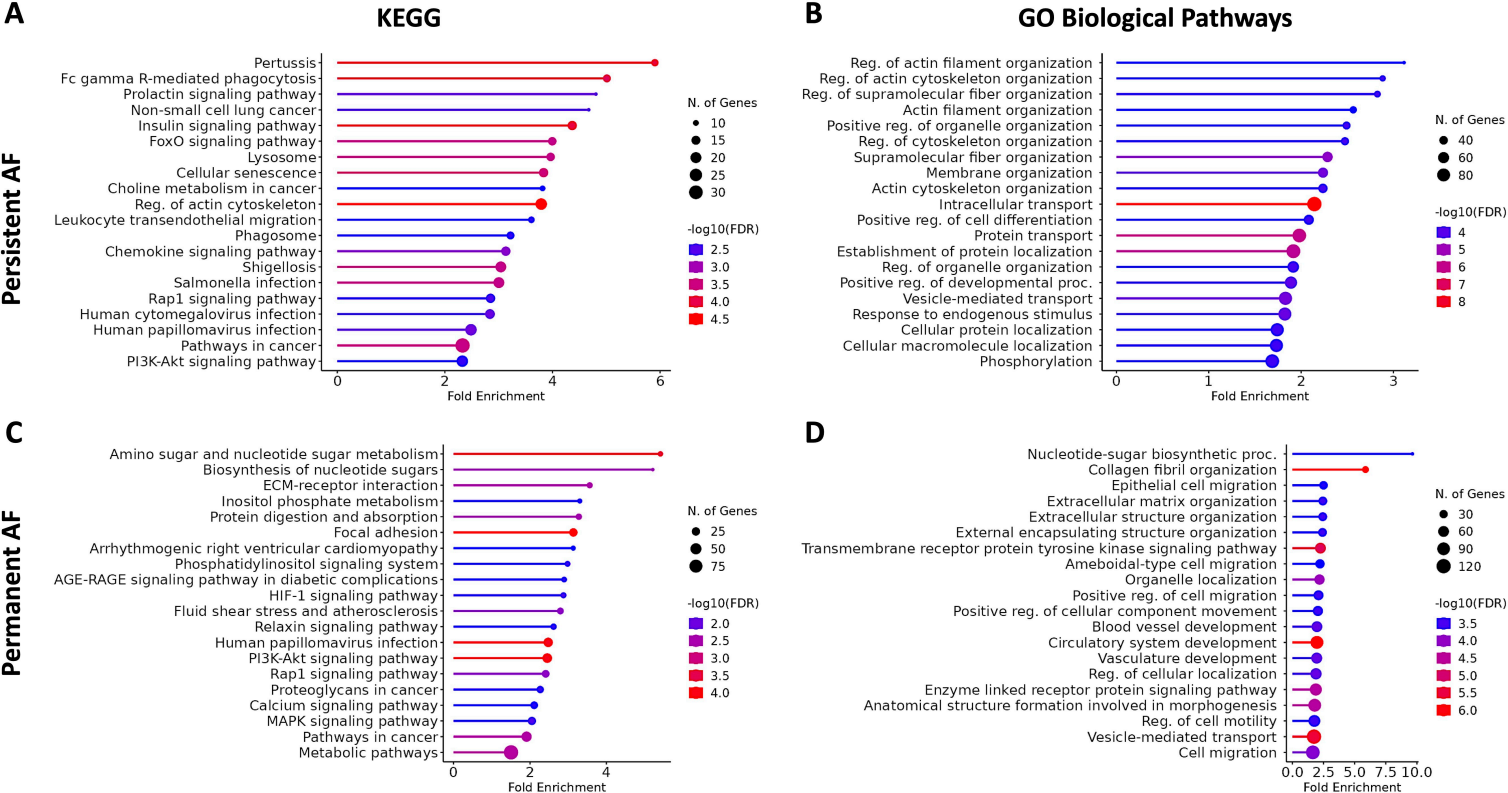
Pathway enrichment analysis of upregulated DEGs in AF. Lollipop plots of enriched KEGG and GO BP pathways in persistent AF (*A,B*) and permanent AF (*C,D*) using ShinyGo. FDR < 0.05. An expanded list of enriched pathways is provided in electronic supplementary material, tables S4–S9.

Upregulated DEGs that were consistently expressed across all datasets were examined in ShinyGo for gene set enrichment analysis. In persistent AF, KEGG and GO BP showed significant enrichment in cellular signalling (figure 4A) and cytoskeletal organization (figure 4B). In permanent AF, metabolic and signalling processes (figure 4C) and extracellular matrix (figure 4D) pathways are significantly enriched. Next, given the differences in immune cell abundances (figures 2 and 3), we further curated specific immune pathways that may be enriched (table 1). Table 1 shows neutrophil, leukocyte and myeloid activation that was present in persistent AF (electronic supplementary material, tables S4–S6). These pathways were not found to be enriched in permanent AF (electronic supplementary material, tables S7–S9). In contrast, neuronal cell development and differentiation were downregulated in persistent AF while molecular metabolic processes were downregulated in permanent AF (electronic supplementary material, figure S1).

**Table 1.** Curated neutrophil-associated pathways in persistent AF.

pathway	enrichment FDR	gene overlap	genes
neutrophil activation	2.92 × 10^−3^	35/597	FUCA2 ITGAL ERP44 HEXB GDI2 RHOA FCGR2B LYZ CMTM6 HSP90AB1 MAPK1 CTSZ MAPK14 QPCT CAT ADGRE5 VAMP7 FGL2 GNS CAB39 ACTR2 DNAJC13 ILF2 IQGAP2 GOLGA7 PTGES2 PSMC3 ITGAM PA2G4 C3AR1 ATP6AP2 NRAS DDX3X CCL5 SURF4
neutrophil-mediated immunity	3.94 × 10^−3^	34/593	FUCA2 ITGAL ERP44 HEXB GDI2 RHOA WDR1 LYZ CMTM6 HSP90AB1 MAPK1 CTSZ MAPK14 QPCT CAT ADGRE5 VAMP7 FGL2 GNS CAB39 ACTR2 DNAJC13 ILF2 IQGAP2 GOLGA7 PTGES2 PSMC3 ITGAM PA2G4 C3AR1 ATP6AP2 NRAS DDX3X SURF4
neutrophil degranulation	2.83 × 10^−3^	31/495	FUCA2 ITGAL ERP44 HEXB GDI2 RHOA LYZ CMTM6 HSP90AB1 MAPK1 CTSZ MAPK14 QPCT CAT ADGRE5 FGL2 GNS CAB39 ACTR2 DNAJC13 ILF2 IQGAP2 GOLGA7 PTGES2 PSMC3 ITGAM PA2G4 C3AR1 ATP6AP2 NRAS DDX3X
granulocyte activation	3.32 × 10^−3^	35/606	FUCA2 ITGAL ERP44 HEXB GDI2 RHOA FCGR2B LYZ CMTM6 HSP90AB1 MAPK1 CTSZ MAPK14 QPCT CAT ADGRE5 VAMP7 FGL2 GNS CAB39 ACTR2 DNAJC13 ILF2 IQGAP2 GOLGA7 PTGES2 PSMC3 ITGAM PA2G4 C3AR1 ATP6AP2 NRAS DDX3X CCL5 SURF4
interleukin-17 signalling	3.69 × 10^−2^	8/76	MAP2K3 MEF2C MAPK1 DUSP3 MAPK14 PPP2CA MAPKAPK3 MAPKAPK2
toll-like receptor 3 TLR3 cascade	4.40 × 10^−2^	9/98	MAP2K3 MEF2C MAPK1 DUSP3 UBE2D3 MAPK14 PPP2CA MAPKAPK3 MAPKAPK2
toll-like receptor cascades	4.40 × 10^−2^	12/158	MAP2K3 MEF2C MAPK1 DUSP3 UBE2D3 MAPK14 PPP2CA MAPKAPK3 CNPY3 CTSK MAPKAPK2 ITGAM
cytokine signalling in the immune system	4.40 × 10^−2^	44/983	CRLF1 GAB2 MAP2K3 PTPN18 PPP2R5C MEF2C DLG3 MAPK1 CSF2RB DUSP3 MAPK14 PPP2CA MAPKAPK3 SOCS2 PIK3CA WDR83 VAMP7 PIN1 UBE2M IL13RA1 LCP1 CASP1 HERC5 IRF8 ARF1 MAPKAPK2 CCR1 YWHAZ PSMC3 ARIH1 STAT3 ITGAM CCL11 CFL1 STAT5B GRB2 MX2 P4HB TNFRSF4 PDGFA CALM1 NRAS IFI30 LYN
myD88-independent TLR4 cascade	4.87 × 10^−2^	9/102	MAP2K3 MEF2C MAPK1 DUSP3 UBE2D3 MAPK14 PPP2CA MAPKAPK3 MAPKAPK2
signalling by VEGF	4.87 × 10^−2^	10/121	CTNNA1 RHOA NRP1 MAPK14 MAPKAPK3 PIK3CA ELMO1 MAPKAPK2 CALM1 NRAS
interleukin-3, interleukin-5 and GM-CSF signalling	4.99 × 10^−2^	7/66	GAB2 CSF2RB PIK3CA YWHAZ STAT5B GRB2 LYN
signalling by interleukins	2.04 × 10^−2^	36/706	CRLF1 GAB2 MAP2K3 PTPN18 PPP2R5C MEF2C DLG3 MAPK1 CSF2RB DUSP3 MAPK14 PPP2CA MAPKAPK3 SOCS2 PIK3CA WDR83 VAMP7 IL13RA1 LCP1 CASP1 ARF1 MAPKAPK2 CCR1 YWHAZ PSMC3 STAT3 ITGAM CCL11 CFL1 STAT5B GRB2 P4HB PDGFA CALM1 NRAS LYN
immune response-regulating cell surface receptor signalling pathway involved in phagocytosis	3.94 × 10^−3^	10/80	FCGR2B HSP90AB1 MAPK1 PIK3CA ACTR2 ELMO1 GRB2 ARPC4 ARPC1A LYN
phagocytosis	6.23 × 10^−3^	21/308	ITGAL FCGR2B ICAM3 TREM2 HSP90AB1 MAPK1 CORO1A PIK3CA VAMP7 ARL8B ACTR2 IRF8 C1orf43 ELMO1 COLEC12 LMAN2 ITGAM GRB2 ARPC4 ARPC1A LYN
Fc gamma R-mediated phagocytosis	2.59 × 10^−4^	13/97	GAB2 FCGR2B MAPK1 PIK3CA ACTR2 CFL2 ARF6 CFL1 RPS6KB2 ARPC4 ARPC1A LYN MARCKS

See electronic supplementary material, table S3, for additional details.

## 4. Discussion

Using microarray and RNAseq bioinformatics approaches, we have shown there is distinct enrichment between DEGs for persistent and permanent AF. Of interest is the difference in expressed immune cell subtypes in atrial tissues when persistent AF tissue is compared to SR control tissues. The increased abundance in myeloid cells can be attributed to the altered immune subtypes, notably in neutrophils where curated pathways for neutrophil activation, neutrophil-mediated immunity and neutrophil degranulation expression levels were higher for persistent AF tissue. In previous studies, increased neutrophil degranulation in atrial tissues was shown to be independent of therapeutics known to influence neutrophil function or systemic inflammation [15]. Interestingly, neutrophil degranulation pathway genes were identified only in persistent AF and were absent in permanent AF. The degranulation of neutrophils may be taken up via resident macrophages, and the degraded neutrophil components within macrophages may sustain the inflammatory microenvironment [16,17]. This suggests the temporal expression of neutrophils in chronic AF is complex [18].

Neutrophils that infiltrate the atrial cross-sectional wall depend on the local atrial vascular endothelium that expresses cytokines and adhesion molecules. For example, polymorphic neutrophils (PMN) express CD11b/CD18 integrins (Mac1), that influence leukocyte extravasation [19–21] and mediate the release of myeloperoxidase (MPO) that adversely affects nitric oxide signalling in the endothelial cells [22]. MPO causes atrial tissue fibrosis and associated AF susceptibility via localized tissue areas of electrical instability and substrates that maintain re-entry pathways [23]. While we have not explored direct changes in MPO expression, increased MPO is associated with neutrophil degranulation, which we observed to be increased in persistent AF.

In our study, the observed increase in neutrophil migration into the atrial tissues in persistent AF corresponds to increased gene expression for pathways related to neutrophil activation and neutrophil immunity. One gene that maps to the pathway for neutrophil activation is CCL5 which is consistently upregulated across all three independent persistent AF datasets. Inhibition of CCL5 in translational models of myocardial infarction [24] with or without coronary reperfusion [25] results in a smaller infarct size with a corresponding significant reduction in neutrophil infiltration [26].

It is presumed that the reduction in neutrophils in persistent AF compared to permanent AF reflects a combination of neutrophils that have undergone apoptosis and non-continued migration to atrial tissues [27]. Neutrophils that undergo apoptotic death secrete a glucocorticoid-inducible protein that inhibits further neutrophil recruitment [27]. Secreted compounds induce MAPK-mediated intracellular signalling, which is enriched in our permanent AF dataset, and is crucial in the regulation of cytoskeletal remodelling and cell adhesion for neutrophils [28]. One study in chronic pressure-overloaded left ventricular mice models has described a role for neutrophils in cardiac apoptosis via ECM degradation and an increase in matrix metalloproteases [29].

Neutrophil apoptosis is complex and can be influenced via the activation of NADPH oxidase as a major source of reactive oxygen species (ROS) production [30,31]. NADPH oxidases, expressed as NOX isoforms, are present in neutrophils and vascular cells, including endothelial cells and pericytes, which are unchanged in persistent AF but are increased in permanent AF. We also found NOX1 was downregulated in persistent AF and NOX4 was upregulated in permanent AF. In translational studies, NOX1 permits vascular migration of endothelial cells [32] which may explain their reduced expression in persistent atrial tissue biopsies. In contrast, NOX4 promotes endothelial angiogenesis that is dependent on eNOS and positive regulation of endothelial cell migration via VEGF-A/VEGFR-2 [33]. In our studies, we observed an increase in VEGFA gene expression and endothelial cell expansion in permanent AF where NOX4 was expressed. The balance between NOX1 and NOX4 expression suggests angiogenesis is promoted when inflammatory signalling is less prominent. In the inflammatory tissue microenvironment, regulatory genes in the PI3K-Akt signalling pathway interfere with angiogenesis progression but also represent a key event in limiting neutrophil survival and self-triggering apoptosis. Neutrophils are downregulated at the end of the persistent phase of AF. Hence, PI3K may have a role in both neutrophil ROS and regulating angiogenesis via angiogenesis mediators released from neutrophil extracellular traps. Interestingly, several anti-angiogenesis genes are also known to be pathogenic in heart failure [34]. The balance of angiogenesis signalling and endothelial function are important mediators in cardiac pathology, including heart failure [34] and AF [35].

Mast cell subtypes were also significantly elevated (resting) and decreased (activated) in persistent AF but not permanent AF. It is well established that mast cell expansion is an early immune response to cardiac tissue inflammation, and their presence is also in part responsible for neutrophil recruitment [36] and the development of AF [37]. However, the presence of increased mast cell density in the myocardium has also been suggested to be protective against fibrosis in CABG surgery patients [38]. This suggests bi-directional effects mediated by mast cells are deleterious in AF patients. Interestingly, our findings concur with the known molecular crosstalk that exists among innate immune cells (e.g. neutrophils and dendritic cells), adaptive immune cells (T cells and B cells) and natural killer cells can contribute to progressive atrial pathology, sustaining AF. In summary, several immune subtypes were perturbed in the persistent stage of AF that were not otherwise present in the permanent stage.

Our study confirmed a decrease in follicular T cells in persistent AF. Follicular T cells have been shown to be associated with AF across several previous studies [39,40], but it is interesting that there is no significant mean increase in permanent AF compared to SR. Neutrophils can activate follicular T cells and promote their local tissue expansion. However, our findings demonstrated that neutrophils but not mean follicular helper T cells were increased in persistent AF tissue. We also demonstrated a mean decrease in the expression of natural killer cells in persistent AF. This observation may also explain why endothelial cells were not elevated in the atrial tissue from pooled persistent AF cases. An increase in natural killer cells may aid the expansion of endothelial cells within the microvascular circulation [41] and promote angiogenesis [42]. Natural killer cells have anti-fibrosis activity [16], and if natural killer cells do not return to baseline, we speculate that fibrosis may progress further in permanent AF.

Downregulated pathways were also identified which differentiate persistent and permanent AF. Persistent AF is associated with downregulation of calcium signalling and neurogenesis pathways, which may be perturbed by fibrotic remodelling, triggering maintenance and reoccurrence of AF [43,44]. In contrast, permanent AF shows downregulation of metabolic processes consistent with a metabolic shift from fatty acid to glucose utilization to increase ATP production efficiency to keep up with oxygen demands [45]. Similar metabolic shifts in cardiac cells have been observed in other end-stage cardiac diseases [46,47] suggesting this may be a common characteristic of prolonged immune-mediated cellular stress. Finally, this study is limited by pooled heterogeneity across multiple independent publicly available datasets. Future studies could verify our identified gene expression changes in relation to phasic immune cell population abundances. In particular, the use of translational animal models of AF could also take advantage of various immune responses that could be controlled across variable time points to determine their precise participation in AF.

In conclusion, we describe the interactions between neutrophils and angiogenesis in human pathological atrial tissue that sustains AF. Angiogenesis is important in cardiac pathology, such as heart failure progression [34]. Our studies have shown distinct changes in the pathological expression of neutrophils and angiogenesis genes in similar but unique phases of chronic AF. Persistent AF appears as a prolonged phase of inflammation, whereas permanent AF is associated with many immune cell abundances returning to control baseline, including neutrophils, mast cells, and natural killer cells but enhanced angiogenesis markers such as endothelial cells and pericytes. However, during the persistent form of AF, there is likely a loss of atrial myocytes and associated atrial fibrosis, which is known to further add to and sustain AF circuits.

## Data Availability

The datasets generated and/or analysed during the current study are available in the Gene Expression Omnibus data repository [48]. Electronic supplementary material is available online [49].

## References

[1] Chiang C, Naditch-Brule L, Murin J, Goethals M, Inoue H, O’Neill J. 2012 Distribution and risk profile of paroxysmal, persistent, and permanent atrial fibrillation in routine clinical practice: insight from the real-life global survey evaluating patients with atrial fibrillation international registry. Circ. Arrhythm. Electrophysiol. **5**, 632–639. (10.1161/CIRCEP.112.970749) 22787011

[2] Suzuki T, Yamazaki T, Ogawa S, Nagai R, Yamashita T, Investigators JRI. 2011 Echocardiographic predictors of frequency of paroxysmal atrial fibrillation (AF) and its progression to persistent AF in hypertensive patients with paroxysmal AF: results from the Japanese Rhythm Management Trial II for Atrial Fibrillation (J-RHYTHM II Study). Heart Rhythm **8**, 1831–1836. (10.1016/j.hrthm.2011.07.035) 21816128

[3] Hammond-Haley M, Providência R, Lambiase PD. 2018 Temporal pattern/episode duration-based classification of atrial fibrillation as paroxysmal vs. persistent: is it time to develop a more integrated prognostic score to optimize management? Europace **20**, f288–f298. (10.1093/europace/eux178) 29016766

[4] Pillarisetti J *et al*. 2009 Evolution of paroxysmal atrial fibrillation to persistent or permanent atrial fibrillation: predictors of progression. J. Atr. Fibrillation **2**, 191. (10.4022/jafib.191) 28496630 PMC5398837

[5] Nso N, Bookani KR, Metzl M, Radparvar F. 2021 Role of inflammation in atrial fibrillation: a comprehensive review of current knowledge. J. Arrhythm. **37**, 1–10. (10.1002/joa3.12473) 33664879 PMC7896450

[6] Jurin I, Hadžibegović I, Durlen I, Jakšić Jurinjak S, Mišković D, Ajduk M, Jerkić H, Letilović T. 2020 Left atrium size and red cell distribution width predict atrial fibrillation progression from paroxysmal or persistent to permanent. Acta Clin. Belg. **75**, 205–211. (10.1080/17843286.2019.1599173) 30950766

[7] Terricabras M, Verma A. 2020 Is pulmonary vein isolation enough for persistent atrial fibrillation? J. Cardiovasc. Electrophysiol. **31**, 2148–2153. (10.1111/jce.14381) 32022320

[8] Noreng S *et al*. 2022 Structure of the core human NADPH oxidase NOX2. Nat. Commun. **13**, 6079. (10.1038/s41467-022-33711-0) 36241643 PMC9568551

[9] Violi F, Carnevale R, Calvieri C, Nocella C, Falcone M, Farcomeni A, Taliani G, Cangemi R, SIXTUS study group. 2015 Nox2 up-regulation is associated with an enhanced risk of atrial fibrillation in patients with pneumonia. Thorax **70**, 961–966. (10.1136/thoraxjnl-2015-207178) 26123660

[10] Khanapure S, Garvey D, Janero D, Gordon Letts L. 2007 Eicosanoids in inflammation: biosynthesis, pharmacology, and therapeutic frontiers. Curr. Top. Med. Chem. **7**, 311–340. (10.2174/156802607779941314) 17305573

[11] Samuchiwal SK, Boyce JA. 2018 Role of lipid mediators and control of lymphocyte responses in type 2 immunopathology. J. Allergy Clin. Immunol. **141**, 1182–1190. (10.1016/j.jaci.2018.02.006) 29477727

[12] Newman AM, Liu CL, Green MR, Gentles AJ, Feng W, Xu Y, Hoang CD, Diehn M, Alizadeh AA. 2015 Robust enumeration of cell subsets from tissue expression profiles. Nat. Methods **12**, 453–457. (10.1038/nmeth.3337) 25822800 PMC4739640

[13] Newman AM *et al*. 2019 Determining cell type abundance and expression from bulk tissues with digital cytometry. Nat. Biotechnol. **37**, 773–782. (10.1038/s41587-019-0114-2) 31061481 PMC6610714

[14] Litviňuková M *et al*. 2020 Cells of the adult human heart. Nature **588**, 466–472. (10.1038/s41586-020-2797-4) 32971526 PMC7681775

[15] Kawasaki M *et al*. 2021 Neutrophil degranulation interconnects over-represented biological processes in atrial fibrillation. Sci. Rep. **11**, 2972. (10.1038/s41598-021-82533-5) 33536523 PMC7859227

[16] Baci D, Bosi A, Parisi L, Buono G, Mortara L, Ambrosio G, Bruno A. 2020 Innate immunity effector cells as inflammatory drivers of cardiac fibrosis. Int. J. Mol. Sci. **21**, 7165. (10.3390/ijms21197165) 32998408 PMC7583949

[17] Lafuse WP, Wozniak DJ, Rajaram MVS. 2020 Role of cardiac macrophages on cardiac inflammation, fibrosis and tissue repair. Cells **10**, 51. (10.3390/cells10010051) 33396359 PMC7824389

[18] Shim HB, Deniset JF, Kubes P. 2022 Neutrophils in homeostasis and tissue repair. Int. Immunol. **34**, 399–407. (10.1093/intimm/dxac029) 35752158 PMC9317988

[19] Ding ZM *et al*. 1999 Relative contribution of LFA-1 and Mac-1 to neutrophil adhesion and migration. J. Immunol. **163**, 5029–5038. (10.4049/jimmunol.163.9.5029) 10528208

[20] Harris ES, McIntyre TM, Prescott SM, Zimmerman GA. 2000 The leukocyte integrins. J. Biol. Chem. **275**, 23409–23412. (10.1074/jbc.r000004200) 10801898

[21] Mócsai A, Ligeti E, Lowell CA, Berton G. 1999 Adhesion-dependent degranulation of neutrophils requires the Src family kinases Fgr and Hck. J. Immunol. **162**, 1120–1126. 9916742

[22] Jerke U, Rolle S, Purfürst B, Luft FC, Nauseef WM, Kettritz R. 2013 β2 integrin-mediated cell-cell contact transfers active myeloperoxidase from neutrophils to endothelial cells. J. Biol. Chem. **288**, 12910–12919. (10.1074/jbc.m112.434613) 23532856 PMC3642334

[23] Rudolph V *et al*. 2010 Myeloperoxidase acts as a profibrotic mediator of atrial fibrillation. Nat. Med. **16**, 470–474. (10.1038/nm.2124) 20305660 PMC2880896

[24] Braunersreuther V *et al*. 2010 Chemokine CCL5/RANTES inhibition reduces myocardial reperfusion injury in atherosclerotic mice. J. Mol. Cell. Cardiol. **48**, 789–798. (10.1016/j.yjmcc.2009.07.029) 19665464

[25] Montecucco F *et al*. 2012 CC chemokine CCL5 plays a central role impacting infarct size and post-infarction heart failure in mice. Eur. Heart J. **33**, 1964–1974. (10.1093/eurheartj/ehr127) 21606075

[26] Carbone F, Nencioni A, Mach F, Vuilleumier N, Montecucco F. 2013 Pathophysiological role of neutrophils in acute myocardial infarction. Thromb. Haemost. **110**, 501–514. (10.1160/TH13-03-0211) 23740239

[27] Ortega‐Gómez A, Perretti M, Soehnlein O. 2013 Resolution of inflammation: an integrated view. EMBO Mol. Med. **5**, 661–674. (10.1002/emmm.201202382) 23592557 PMC3662311

[28] Greenlee‐Wacker MC. 2016 Clearance of apoptotic neutrophils and resolution of inflammation. Immunol. Rev. **273**, 357–370. (10.1111/imr.12453) 27558346 PMC5000862

[29] Kolpakov MA *et al*. 2009 Pleiotropic effects of neutrophils on myocyte apoptosis and left ventricular remodeling during early volume overload. J. Mol. Cell. Cardiol. **47**, 634–645. (10.1016/j.yjmcc.2009.08.016) 19716828 PMC2761535

[30] El Kebir D, Filep JG. 2013 Modulation of neutrophil apoptosis and the resolution of inflammation through β2 integrins. Front. Immunol. **4**, 60. (10.3389/fimmu.2013.00060) 23508943 PMC3589696

[31] Kunes P, Krejsek J, Brtko M, Mandak J, Kolackova M, Trojackova Kudlova M, Andrys C. 2009 Neutrophil apoptosis by Fas/FasL: harmful or advantageous in cardiac surgery? Thorac. Cardiovasc. Surg. **57**, 1–6. (10.1055/s-2008-1039060) 19169988

[32] Fernandes DC, Wosniak J, Gonçalves RC, Tanaka LY, Fernandes CG, Zanatta DB, de Mattos ABM, Strauss BE, Laurindo FRM. 2021 PDIA1 acts as master organizer of NOX1/NOX4 balance and phenotype response in vascular smooth muscle. Free Radic. Biol. Med. **162**, 603–614. (10.1016/j.freeradbiomed.2020.11.020) 33227407

[33] Craige SM *et al*. 2011 NADPH oxidase 4 promotes endothelial angiogenesis through endothelial nitric oxide synthase activation. Circulation **124**, 731–740. (10.1161/circulationaha.111.030775) 21788590 PMC3589548

[34] Shiojima I, Sato K, Izumiya Y, Schiekofer S, Ito M, Liao R, Colucci WS, Walsh K. 2005 Disruption of coordinated cardiac hypertrophy and angiogenesis contributes to the transition to heart failure. J. Clin. Invest. **115**, 2108–2118. (10.1172/JCI24682) 16075055 PMC1180541

[35] Corban MT, Toya T, Ahmad A, Lerman LO, Lee HC, Lerman A. 2021 Atrial fibrillation and endothelial dysfunction: a potential link? Mayo Clin. Proc. **96**, 1609–1621. (10.1016/j.mayocp.2020.11.005) 33775421

[36] Uemura K *et al*. 2016 Mast cells play an important role in the pathogenesis of hyperglycemia‐induced atrial fibrillation. J. Cardiovasc. Electrophysiol. **27**, 981–989. (10.1111/jce.12995) 27097848

[37] Liao C hui *et al*. 2010 Cardiac mast cells cause atrial fibrillation through PDGF-A-mediated fibrosis in pressure-overloaded mouse hearts. J. Clin. Invest. **120**, 242–253. (10.1172/JCI39942) 20038802 PMC2798688

[38] Legere SA, Haidl ID, Castonguay MC, Brunt KR, Légaré JF, Marshall JS, IMPART Investigator Team Canada. 2020 Increased mast cell density is associated with decreased fibrosis in human atrial tissue. J. Mol. Cell. Cardiol. **149**, 15–26. (10.1016/j.yjmcc.2020.09.001) 32931784

[39] Jiang F, Zhang W, Lu H, Tan M, Zeng Z, Song Y, Ke X, Lin F. 2022 Prediction of herbal medicines based on immune cell infiltration and immune- and ferroptosis-related gene expression levels to treat valvular atrial fibrillation. Front. Genet. **13**, 886860. (10.3389/fgene.2022.886860) 36246656 PMC9554472

[40] Wang X, Fan H, Wang Y, Yin X, Liu G, Gao C, Li X, Liang B. 2021 Elevated peripheral T helper cells are associated with atrial fibrillation in patients with rheumatoid arthritis. Front. Immunol. **12**, 744254. (10.3389/fimmu.2021.744254) 34721413 PMC8554094

[41] Ayach BB *et al*. 2006 Stem cell factor receptor induces progenitor and natural killer cell-mediated cardiac survival and repair after myocardial infarction. Proc. Natl Acad. Sci. USA **103**, 2304–2309. (10.1073/pnas.0510997103) 16467148 PMC1413746

[42] Sun K, Li Y yuan, Jin J. 2021 A double-edged sword of immuno-microenvironment in cardiac homeostasis and injury repair. Signal Transduct. Target. Ther. **6**, 79. (10.1038/s41392-020-00455-6) 33612829 PMC7897720

[43] Steenman M. 2020 Insight into atrial fibrillation through analysis of the coding transcriptome in humans. Biophys. Rev. **12**, 817–826. (10.1007/s12551-020-00735-z) 32666467 PMC7429641

[44] Huang J, Wu B, Qin P, Cheng Y, Zhang Z, Chen Y. 2023 Research on atrial fibrillation mechanisms and prediction of therapeutic prospects: focus on the autonomic nervous system upstream pathways. Front. Cardiovasc. Med. **10**, 1270452. (10.3389/fcvm.2023.1270452) 38028487 PMC10663310

[45] Qin X, Zhang Y, Zheng Q. 2022 Metabolic inflexibility as a pathogenic basis for atrial fibrillation. Int. J. Mol. Sci. **23**, 8291. (10.3390/ijms23158291) 35955426 PMC9368187

[46] Taylor J *et al*. 2023 Transcriptomic comparison of human peripartum and dilated cardiomyopathy identifies differences in key disease pathways. J. Cardiovasc. Dev. Dis. **10**, 188. (10.3390/jcdd10050188) 37233155 PMC10218903

[47] Hoes MF, Bomer N, Ricke-Hoch M, de Jong TV, Arevalo Gomez KF, Pietzsch S, Hilfiker-Kleiner D, van der Meer P. 2020 Human iPSC-derived cardiomyocytes of peripartum patients with cardiomyopathy reveal aberrant regulation of lipid metabolism. Circulation **142**, 2288–2291. (10.1161/circulationaha.119.044962) 33284656 PMC7846285

[48] NCBI. Gene Expression Omnibus. See https://www.ncbi.nlm.nih.gov/geo/.

[49] Stone E, Taylor J, Li A, McLachlan CS. 2025 Supplementary material from: Transcriptional meta-analysis and immune cell profiles reveals altered neutrophil dynamics in chronic atrial fibrillation. FigShare (10.6084/m9.figshare.c.7699003) PMC12311809 40747357

